# Magnetic resonance imaging detects significant sex differences in human myocardial strain

**DOI:** 10.1186/1475-925X-10-76

**Published:** 2011-08-22

**Authors:** Jennifer S Lawton, Brian P Cupps, Andrew K Knutsen, Ningning Ma, Beckah D Brady, Lina M Reynolds, Michael K Pasque

**Affiliations:** 1 Department of Surgery, Washington University School of Medicine, 660 S. Euclid Ave., St. Louis, Missouri, 63110, USA

## Abstract

**Background:**

The pathophysiology responsible for the significant outcome disparities between men and women with cardiac disease is largely unknown. Further investigation into basic cardiac physiological differences between the sexes is needed. This study utilized magnetic resonance imaging (MRI)-based multiparametric strain analysis to search for sex-based differences in regional myocardial contractile function.

**Methods:**

End-systolic strain (circumferential, longitudinal, and radial) was interpolated from MRI-based radiofrequency tissue tagging grid point displacements in each of 60 normal adult volunteers (32 females).

**Results:**

The average global left ventricular (LV) strain among normal female volunteers (n = 32) was significantly larger in absolute value (functionally better) than in normal male volunteers (n = 28) in both the circumferential direction (Male/Female = -0.19 ± 0.02 vs. -0.21 ± 0.02; p = 0.025) and longitudinal direction (Male/Female = -0.14 ± 0.03 vs. -0.16 ± 0.02; p = 0.007).

**Conclusions:**

The finding of significantly larger circumferential and longitudinal LV strain among normal female volunteers suggests that baseline contractile differences between the sexes may contribute to the well-recognized divergence in cardiovascular disease outcomes. Further work is needed in order to determine the pathologic changes that occur in LV strain between women and men with the onset of cardiovascular disease.

## Background

Cardiovascular disease is the number one cause of death in the United States. Each year, since 1984, more women than men have died from this disease [1]. Women are more likely to die following their first heart attack, are more likely to die following coronary artery bypass surgery, and are more likely to present with sudden death when compared to men [2-8]. The most promising avenue to understanding these clinical outcome disparities between men and women with cardiac disease is to investigate sex-related differences in basic physiological mechanisms such as those involved in the regional contractile function of the heart.

Recent investigations into sex-based differences in myocardial contractile function have utilized a broad spectrum of imaging methodologies and have demonstrated markedly conflicting results in this foundational area of basic cardiac physiology [9-17]. Cardiac MRI with radiofrequency tissue tagging has demonstrated the highest spatial and temporal resolution in the three-dimensional tracking of the *intramyocardial *point displacements that are utilized to generate 3D left ventricular strain maps [18-22].

## Methods

### Patient Characteristics

Sixty normal volunteers (age 33.1 ± 10.8 years) with no history of any cardiac disease underwent MRI-based multiparametric strain analysis. The study group consisted of 32 women and 28 men. No significant differences were found between the two groups in age (women: 34.8 ± 10.4 vs. men: 31.6 ± 11.3 years), systolic blood pressure (women: 117.0 ± 10.4 vs. men: 123.6 ± 15.0 mmHg), or diastolic blood pressure (women: 73.7 ± 7.9 vs. men: 73.6 ± 11.4 mmHg). The Human Research Protection Office at Washington University, St. Louis, MO, approved the study and all subjects gave informed written consent.

### MR Imaging

Imaging was performed in a 1.5 Tesla MR scanner (Siemens Medical Systems, Malvern, PA). Multiple short axis image sets were acquired in parallel planes at 8 mm intervals extending from the plane of the mitral valve to the apex of the heart. Additionally, four sets of long axis images oriented radially and intersecting the centroid of the ventricle were also obtained. For each selected imaging plane, a single-slice MR tagged image was collected with a sequence consisting of a spatial modulation of magnetization (SPAMM) radiofrequency tissue-tagging preparation [23,24] followed by a 2-D balanced steady-state free precession (SSFP) cine image acquisition. Image acquisition was synchronized with real-time electrocardiogram (ECG) at the time of the MRI scanning. Typical imaging parameters were: tag spacing 8 mm; slice thickness 8 mm; repetition time 30.3 ms, echo time 2.2 ms, field of view 306 × 350 mm and image matrix 168 × 256.

### Strain Calculations

**Figure 1:**
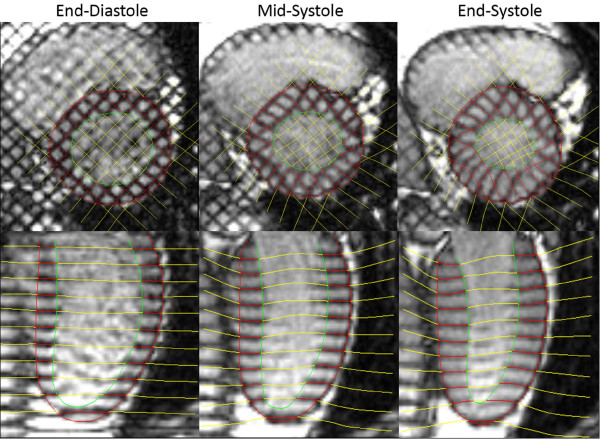
**MRI Motion Tracking**. Tagged MRI images of the left ventricle are shown at end-diastole, mid-systole, and end-systole. Images are acquired approximately every 30 ms throughout the complete heart cycle. Tag lines are semi-automatically tracked through systole, and displacements are computed using a previously described and validated method [20].

The method used to compute strain in this study has been described previously [20], so only a brief summary is provided here. Manually identified endocardial and epicardial boundaries from the tagged MR images were used to construct a finite element model of the LV. Utilizing the anterior and posterior junction points between the right ventricle (RV) free wall and intraventricular septum as anatomical markers, a mesh of twelve hexahedral elements (anterior, anterolateral, posterolateral, posterior, posteroseptal and anteroseptal myocardial walls) at the base and mid-LV regions, and six pentahedral elements at the apex, was constructed. Three-dimensional systolic displacements were computed from the deformation of the tag surfaces (Figure 1) using a previously described and validated method [20]. Analysis of the displacement data was carried out in the finite element analysis software package StressCheck (ESRD, Inc., St. Louis, Missouri). A continuous representation of displacement at any point within the domain of the model was obtained from a least squares fitting of the measured displacements. Values of circumferential, longitudinal and radial strain were computed for an evenly spaced grid in the element coordinate system (15,300 total points). These point-wise measurements were used to compute regional average strain values for the anterior, anterolateral, posterolateral, posterior, posteroseptal and anteroseptal walls in each of the sixty volunteers. Additionally, average values in both men and women for each of the three strain components were computed at each of the 15,300 points covering the LV. The differences between these average strain values at corresponding points were used to generate color contour plots illustrating differences in contractile function throughout the LV in men and women.

### Statistical Analyses

All strain values are expressed as mean ± standard deviation. Regional and full model strain comparisons between men and women were done using an unpaired t-test. P-values were adjusted for multiple comparisons using the Dunn-Sidak correction. Normality of the strain data was tested using the Shapiro-Wilk statistic. In all cases, a p-value of 0.05 was considered to be significant. Statistical analyses were carried out using the Systat software package (Systat Software, Inc., Chicago, IL).

In sample size determination, an effect size of .75 was estimated based on strain differences between male and females. A two-sided unpaired t-test sample size calculation with alpha = .05 produced a power of .81 if the two groups had 30 patients each. When all data was gathered, circumferential strain differences between males and females produced an effect size of one. Thus, in a post-hoc power calculation with 28 males and 32 females, the achieved power with alpha = .05 was .97. (G*Power 3.1.2). Age does not appear to be a significant variable in systolic strain levels [13] in the relatively narrow age range of our study group (33.3 ± 10.8 years).

## Results

**Figure 2:**
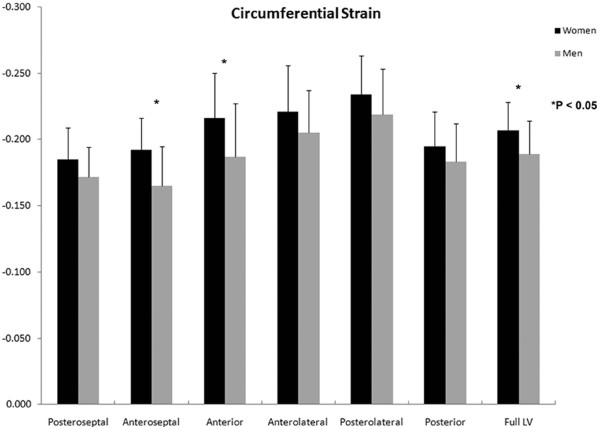
**Regional Circumferential Strain Comparison between Men and Women**. Circumferential strain means with standard deviation bars of men and women are demonstrated. Adjusted p-values are as follows: Posteroseptal, p = .224; Anteroseptal, p = .002; Anterior, p = .027; Anterolateral, p = .439; Posterolateral, p = .394; Posterior, p = .542; Full LV, p = .025. Circumferential strain values were significantly greater in women than men both globally and in the anterior and anteroseptal walls.

**Figure 3:**
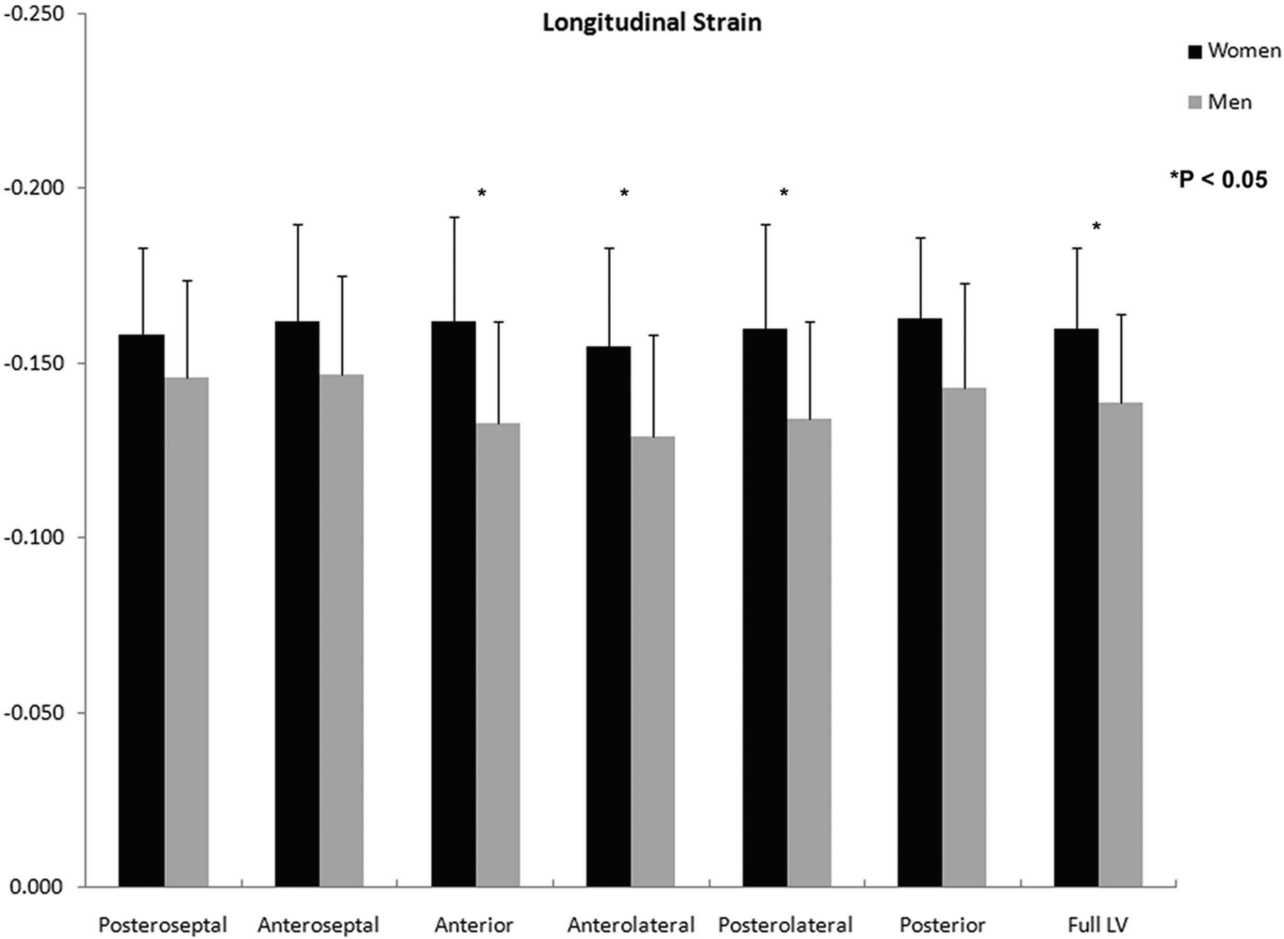
**Regional Longitudinal Strain Comparison between Men and Women**. Longitudinal strain means (with standard deviation bars) of men and women are demonstrated. Adjusted p-values are as follows: Posteroseptal, p = .500 Anteroseptal, p = .237; Anterior, p = .004; Anterolateral, p = .005; Posterolateral, p = .006; Posterior, p = .041; Full LV, p = .007. Significant differences in longitudinal strain between the sexes were found globally and in the anterior, anterolateral and posterolateral walls.

**Figure 4:**
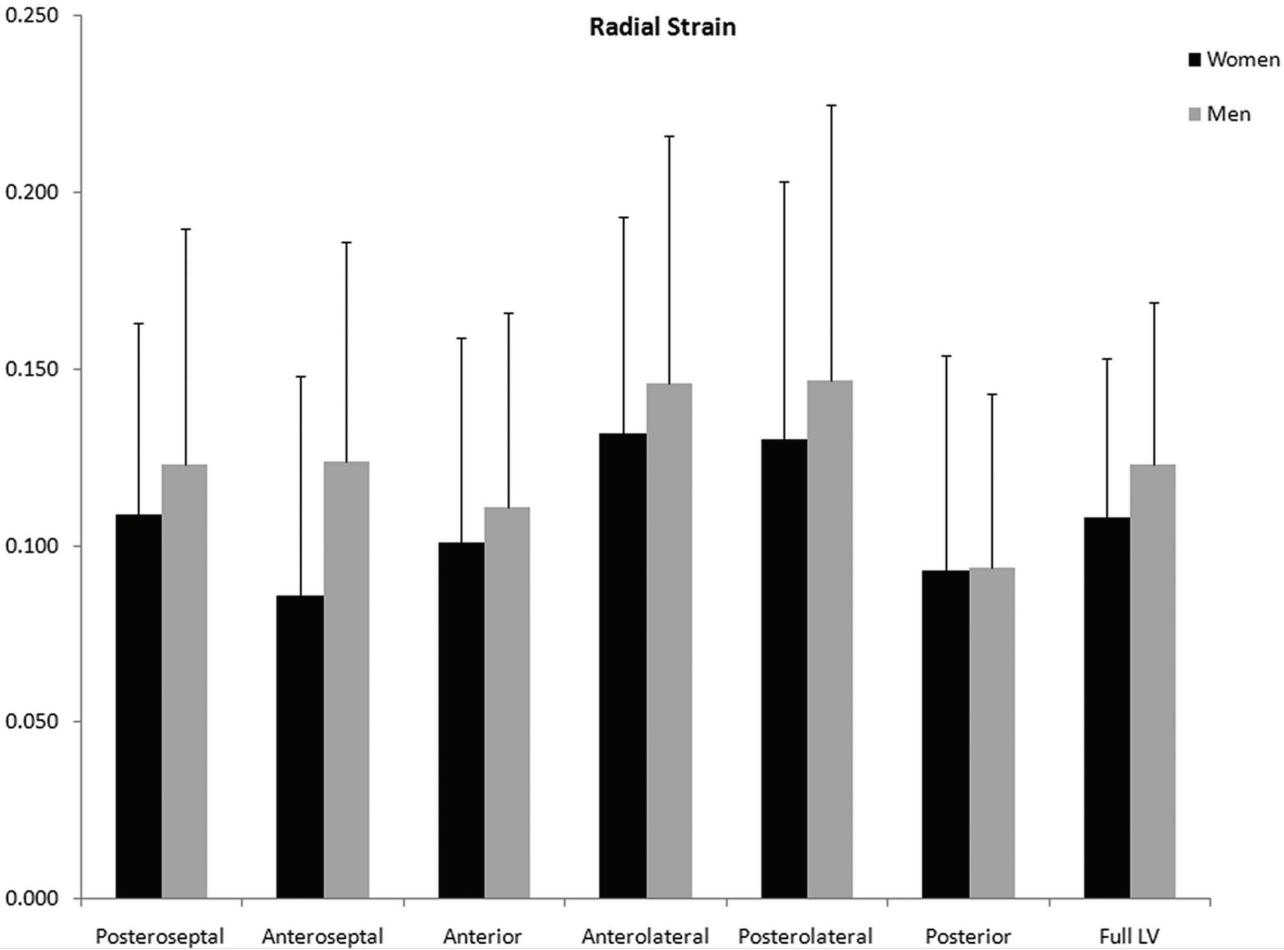
**Regional Radial Strain Comparison between Men and Women**. Radial strain means (with standard deviation bars) of men and women are demonstrated. Adjusted p-values are as follows: Posteroseptal, p = .964; Anteroseptal, p = .140; Anterior, p = .995; Anterolateral, p = .966; Posterolateral, p = .968; Posterior, p = 1.000; Full LV, p = .789. No significant differences in regional or global radial strain values were observed between men and women.

**Figure 5:**
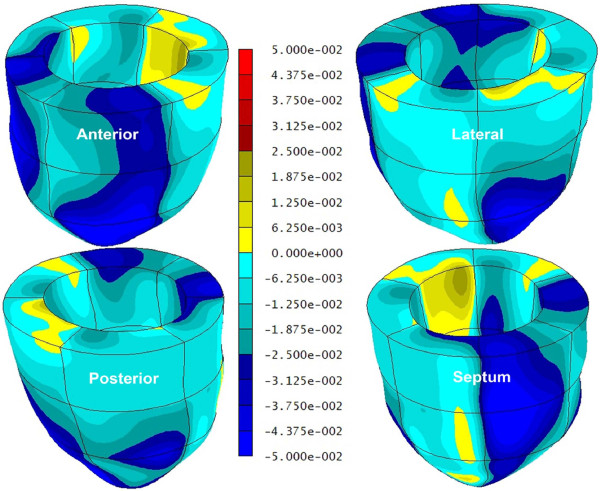
**Circumferential Strain Contour Plots in Men and Women**. This left ventricular color contour plot graphically demonstrates the differences in *circumferential *strain between men and women. Average strain values for both groups were computed for a grid of 15,300 points within the ventricle. The *differences *between the male and female average values at corresponding points were used to generate the contour plot in this figure. All shades of blue in the plot indicate regions where strain values were larger (in absolute value) in women, while yellow and red colors indicate regions where strain values were larger in men.

**Figure 6:**
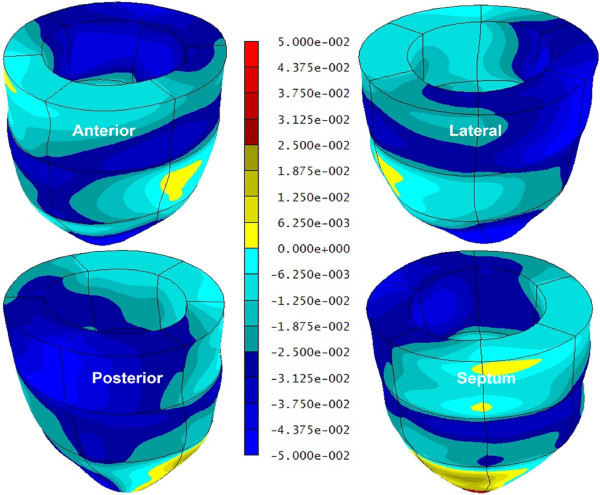
**Longitudinal Strain Contour Plots in Men and Women**. This left ventricular color contour plot graphically demonstrates the differences in *longitudinal *strain between men and women. Average strain values for both groups were computed for a grid of 15,300 points within the ventricle. The *differences *between the male and female average values at corresponding points were used to generate the contour plot in this figure. All shades of blue in the plot indicate regions where strain values were larger (in absolute value) in women, while yellow and red colors indicate regions where strain values were larger in men.

**Figure 7:**
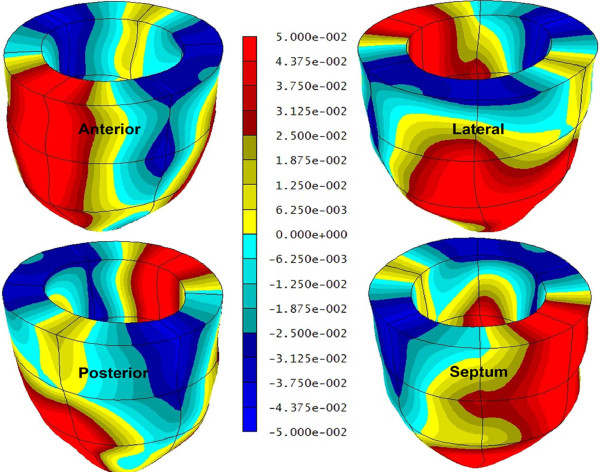
**Radial Strain Contour Plots in Men and Women**. This left ventricular color contour plot graphically demonstrates the differences in *radial *strain between men and women. Average strain values for both groups were computed for a grid of 15,300 points within the ventricle. The *differences *between the male and female average values at corresponding points were used to generate the contour plot in this figure. All shades of blue in the plot indicate regions where radial strain values were larger in women, while yellow and red colors indicate regions where radial strain values in men exceeded those in women. There is no statistically significant difference in radial strain between men and women when compared on a regional or global left ventricular basis.

**Figure 8:**
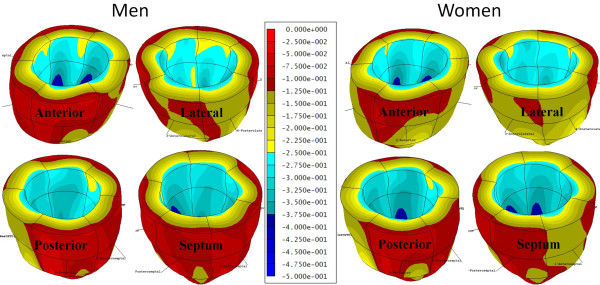
**Mean Circumferential Maps in Men and Women**. These left ventricular color contour plots graphically demonstrate circumferential strain averaged across the 28 males (left), and 32 females (right).

**Figure 9:**
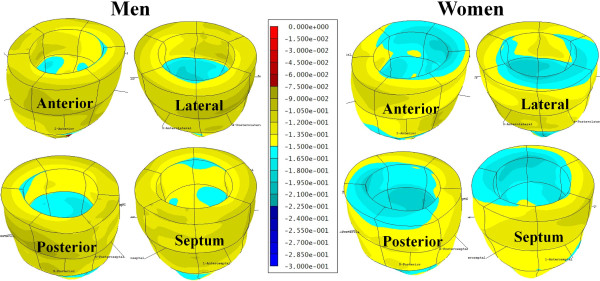
**Mean Longitudinal Strain Maps in Men and Women**. These left ventricular color contour plots graphically demonstrate longitudinal strain averaged across the 28 males (left), and 32 females (right).

**Figure 10:**
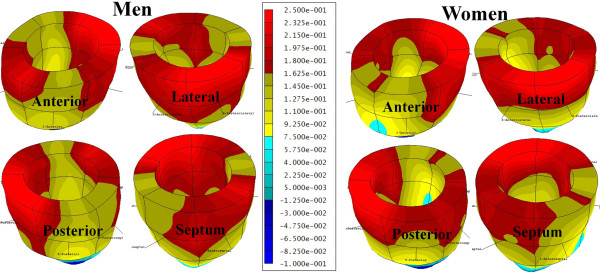
**Mean Radial Strain Maps in Men and Women**. These left ventricular color contour plots graphically demonstrate radial strain averaged across the 28 males (left), and 32 females (right).

A significant sex-based difference in two of three major components of regional myocardial strain was demonstrated, including a 10% reduction (in absolute value) in global male *circumferential *strain compared to female: M/F = -0.19 ± 0.02 vs. -0.21 ± 0.02 (p = 0.025). Similarly, global *longitudinal *strain comparison demonstrated a 13% male reduction versus female values: M/F = -0.14 ± 0.03 vs. -0.16 ± 0.02 (p = 0.007). Radial strain comparison demonstrated no significant difference between the sexes with the expected wide standard deviations: M/F = 0.12 ± 0.05 vs. 0.11 ± 0.05 (p = 0.789). Several significant differences in regional circumferential and longitudinal strain were seen between men and women (Figures 2 and 3, respectively), while no significant differences in regional radial strain were observed (Figure 4). Point-wise differences in average values of each strain component between men and women were used to create color contour plots to provide a visual illustration of baseline differences in contractile function between the sexes throughout the LV. These plots are presented in Figures 5, 6 and 7. Mean strain maps of each strain component are also demonstrated for both males and females (Figures 8, 9 and 10).

## Discussion

Myocardial systolic strain is the foundational index in the quantification of regional myocardial contractile function. That there would be a significant sex-based difference in the contractile function of human myocardium at this most basic level is not intuitive. Nonetheless, this application of advanced mathematical analyses to the highly accurate spatial and temporal left ventricular geometrical data supplied by magnetic resonance tracking of radiofrequency tag planes in the in vivo human myocardium has characterized a foundational sex-based difference in myocardial mechanical function at its most basic level. The contractile information supplied by the application of these advanced engineering analyses in the clinical setting is the foundation upon which future diagnostic and therapeutic algorithms may well be based [25-27]. The establishment of a *normal human strain database*, as characterized in this study, is critical to any attempts to *normalize *strain data since all components of myocardial strain are geometrically heterogeneous and now, as a result of this investigation, must also be normalized based on sex. The clinical implications of the availability of a geometrical and sex differentiated normal human strain database are potentially profound. Such a database enables the accurate normalization of patient-specific systolic strain components thus uniquely allowing these strain indices (with often widely variable normal data ranges) to be combined into powerful composite multiparametric strain analyses to accurately characterize in vivo human left ventricular regional and transmural myocardial contractile function [28].

Previous studies on strain differences between the sexes have reported conflicting results. In 2009, Neizel found no significant difference in peak circumferential strains between the sexes utilizing SENC imaging [10]. Similar to our study, Hurlburt reported significant differences in both circumferential and longitudinal strain between men and women using speckle-tracking echocardiography [11]. In 2011, Reckefuss found longitudinal strain to be significantly higher in women but no difference between the sexes in radial strain [12]. Kuznetsova reported radial strain being significantly higher in women than in men, but longitudinal strain having no significant differences [17]. Sun found no sex-related strain differences [13]. Several studies have investigated the relationship between MRI measured strains and other parameters utilizing data collected as part the Multi-Ethnic Study of Atherosclerosis (MESA) [14-16]. In 2005, Rosen and colleagues found that the relationship between peak systolic circumferential strain and the ratio of LV mass to end-diastolic volume is significantly modified by gender [16]. In a subsequent manuscript, Rosen reported a significant relationship between CRP levels and regional LV function in men, but found no association in women [15]. Interestingly, in another study using data from MESA participants, Fernandes reported a direct significant relationship between ejection fraction and circumferential strain that did not differ by gender [14]. Previously, noninvasive assessment of cardiac function in women has suggested that they have higher baseline ejection fraction compared to men [9]. Clearly, there is significant discord in the reported results of clinical investigation into sex-based differences in myocardial strain.

Significantly, all of these previous studies were *two-dimensional *in nature. That is, the regional strain values from these studies were based on measurements from a single imaging plane. In contrast, the method used in the present investigation combines data from multiple planes to generate three-dimensional displacements that more accurately reflect the true myocardial contractile motion of the left ventricle. Of equal importance, the data from this study provides an atlas of strain values from men and women that is generated from the cardiac imaging modality with the highest spatial and temporal resolution. This database information is retained in a format that can be used to generate sex-based normalized measures of contractile function in different patient populations. This data provides a springboard from which investigators can fully characterize sex differences in contractile function and understand their role in heart disease.

Further strengths of our multi-parametric strain approach are found in the use of a *p*-version finite element model to estimate strain in the left ventricle. A least-squares fit of each component of the displacement vectors is computed over the elements of the mesh. The result is a continuous distribution of 3D strain estimates over the entire left ventricle. This is in contrast to a more common approach of computing derivatives on a grid of points using finite differences. We acknowledge that some limitations do exist in our approach. The calculated fits and strain estimates are only as good as the displacement vectors obtained from the imaging data. Image noise and patient movement between breath holds can introduce error into the measured displacement vectors. The fact that the significant differences in strain were demonstrated in our investigation only in the primary directions of myocyte contraction (circumferential and longitudinal) is not surprising. Measurements of *radial *strain from tagged MR images tend to be more variable than similarly obtained measurements of circumferential or longitudinal strain [29]. It has been postulated that this variability is in part attributable to the reduced density of tag lines in the radial direction typically available on tagged MR images [30]. The variability of the calculated radial strain values likely contributes to the lack of observed significant differences in radial strain between the sexes seen in this study.

## Conclusions

Our data suggest that at least prior to the development of cardiovascular disease, women have greater systolic strain than their male counterparts. The assumption that investigations into the regional contractile function of the heart can be referenced to the normal average and standard deviation of the general population is no longer appropriate. The sex-based differences in systolic myocardial strain that have been established by this investigation must be accounted for in future investigations of regional contractile function. Fundamental to any such clinical investigation is the availability of a normal human strain database (such as that utilized by our laboratory in this investigation) that allows the normalization of patient-specific strain to the appropriate sex-based normal average and standard deviation. The normal human left ventricular strain information from 60 normal volunteers that was utilized in this investigation is available upon request from our laboratory.

## Competing interests

Authors Michael K. Pasque and Brian P. Cupps have a financial interest related to the intellectual property of the computer software developed and used in this research study through the company CardioWise, LLC. Dr. Pasque, Dr. Cupps, and Washington University may receive income based on a license of related technology by the University to CardioWise, LLC. CardioWise, LLC did not support this work. All remaining authors, including Dr. Jennifer S. Lawton, Andrew K. Knutsen, Ningning Ma, Beckah D. Brady and Lina M. Reynolds have no competing interests.

## Authors' contributions

JL conceived the study, participated in its design and data analysis and drafted the manuscript. MP directs the laboratory and participated directly in the data analysis, statistical approach, and manuscript design and crafting. BC participated in image acquisition, image analysis, data analysis and statistical approach. AK, NM and BB assisted in image analysis, data analysis and statistical approach. LR assisted in image data acquisition, regulatory management, and data analysis and management. Final approval of the final version of the manuscript was given by all authors.
